# Supplementary material for: CRUMBLER: A tool for the prediction of ancestry in cattle
Source: PLoS One. 2019 Aug 26;14(8):e0221471. doi: 10.1371/journal.pone.0221471 (PMC6709893; doi:10.1371/journal.pone.0221471)
Supplement: S1 Fig — (PDF) [file pone.0221471.s003.pdf]

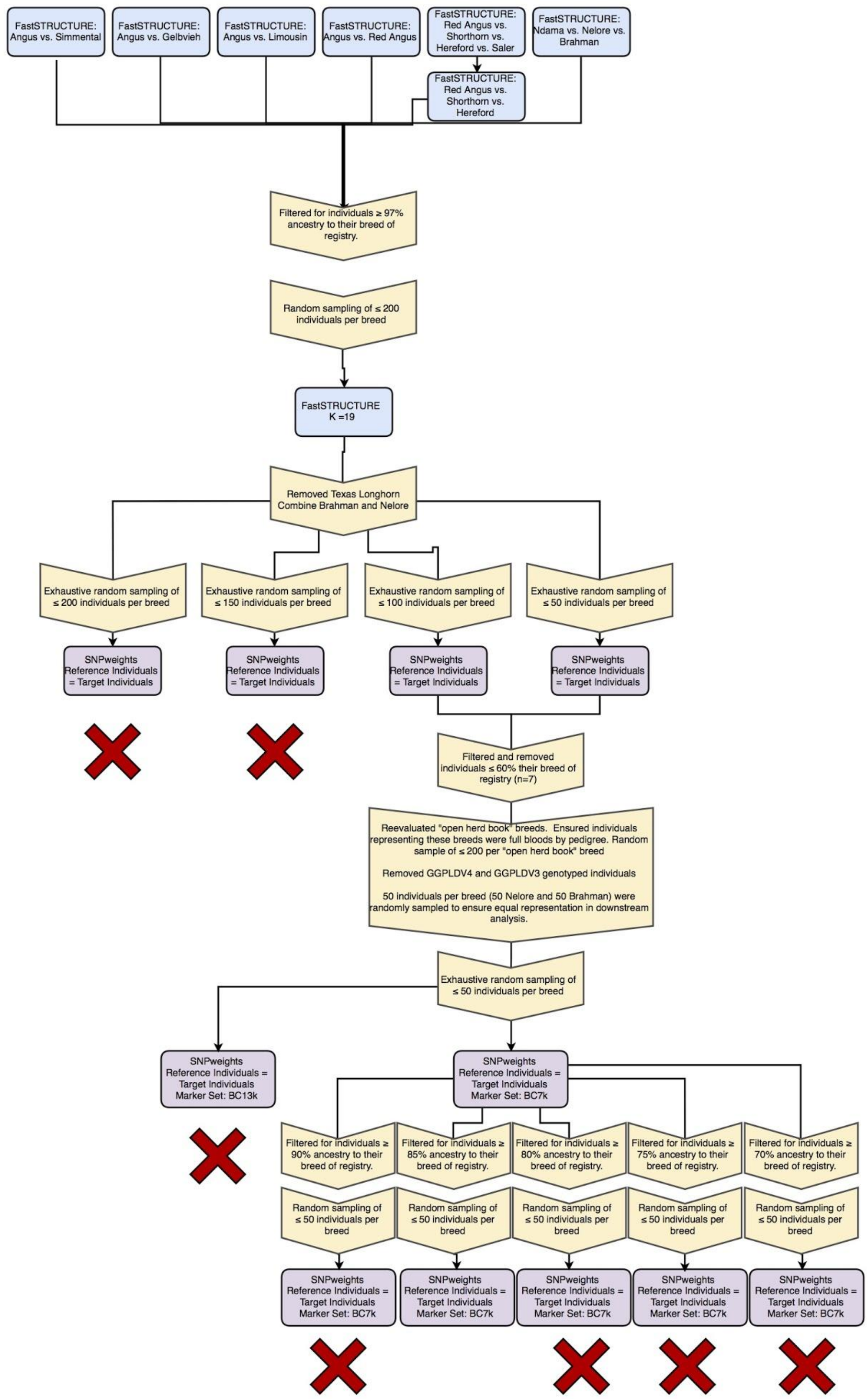

S1 Fig. An overview of the processes and iterations of filtering conducted in the development of the reference panel. Blue = FastSTRUCTURE analyses, Purple = SNPweights analyses, Yellow Arrows = Data management processes.
