# Supplementary material for: CRUMBLER: A tool for the prediction of ancestry in cattle
Source: PLoS One. 2019 Aug 26;14(8):e0221471. doi: 10.1371/journal.pone.0221471 (PMC6709893; doi:10.1371/journal.pone.0221471)
Supplement: S3 Fig — (PDF) [file pone.0221471.s005.pdf]

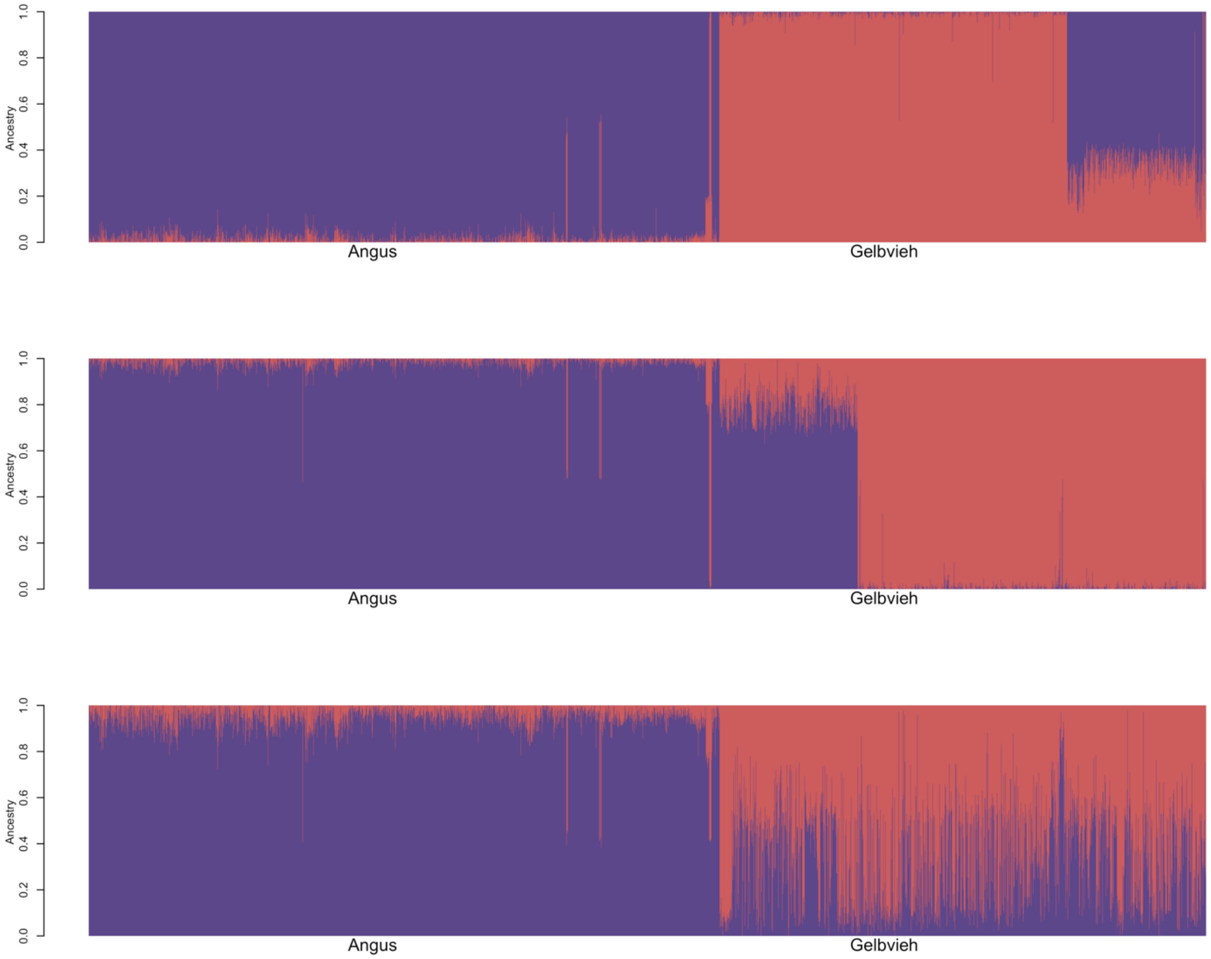

S3 Fig. Preliminary FastSTRUCTURE analysis of candidate Angus and Gelbvieh reference population animals. Three analyses were run to compare equal numbers of animals from each breed when the available sample sizes for each breed differed. Each row represents an analysis and each animal is represented as a vertical line.
