# Supplementary material for: CRUMBLER: A tool for the prediction of ancestry in cattle
Source: PLoS One. 2019 Aug 26;14(8):e0221471. doi: 10.1371/journal.pone.0221471 (PMC6709893; doi:10.1371/journal.pone.0221471)
Supplement: S6 Fig — (PDF) [file pone.0221471.s008.pdf]

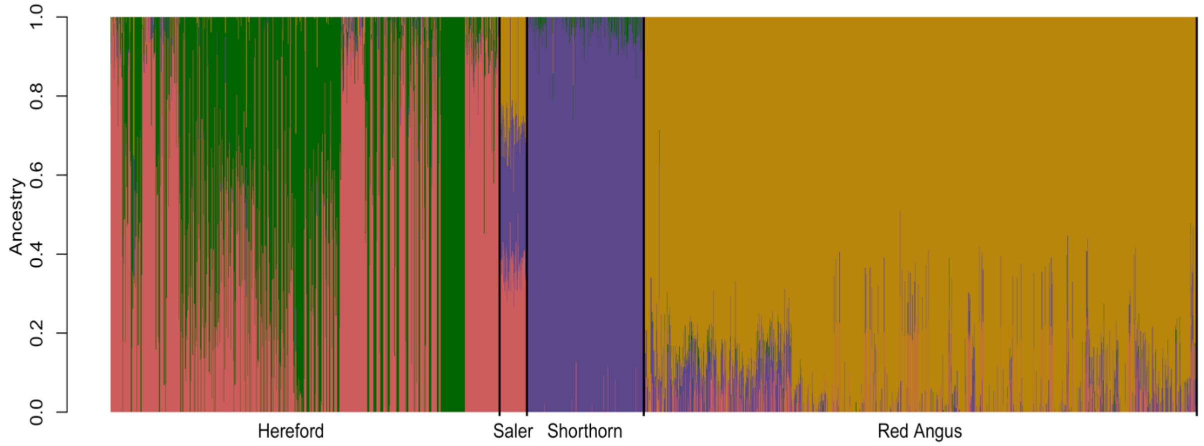

S6 Fig. Preliminary FastSTRUCTURE analysis of candidate Red Angus, Hereford, Shorthorn, and Salers reference population animals. Each animal is represented as a vertical line.
