# Supplementary material for: CRUMBLER: A tool for the prediction of ancestry in cattle
Source: PLoS One. 2019 Aug 26;14(8):e0221471. doi: 10.1371/journal.pone.0221471 (PMC6709893; doi:10.1371/journal.pone.0221471)
Supplement: S10 Fig — (PDF) [file pone.0221471.s012.pdf]

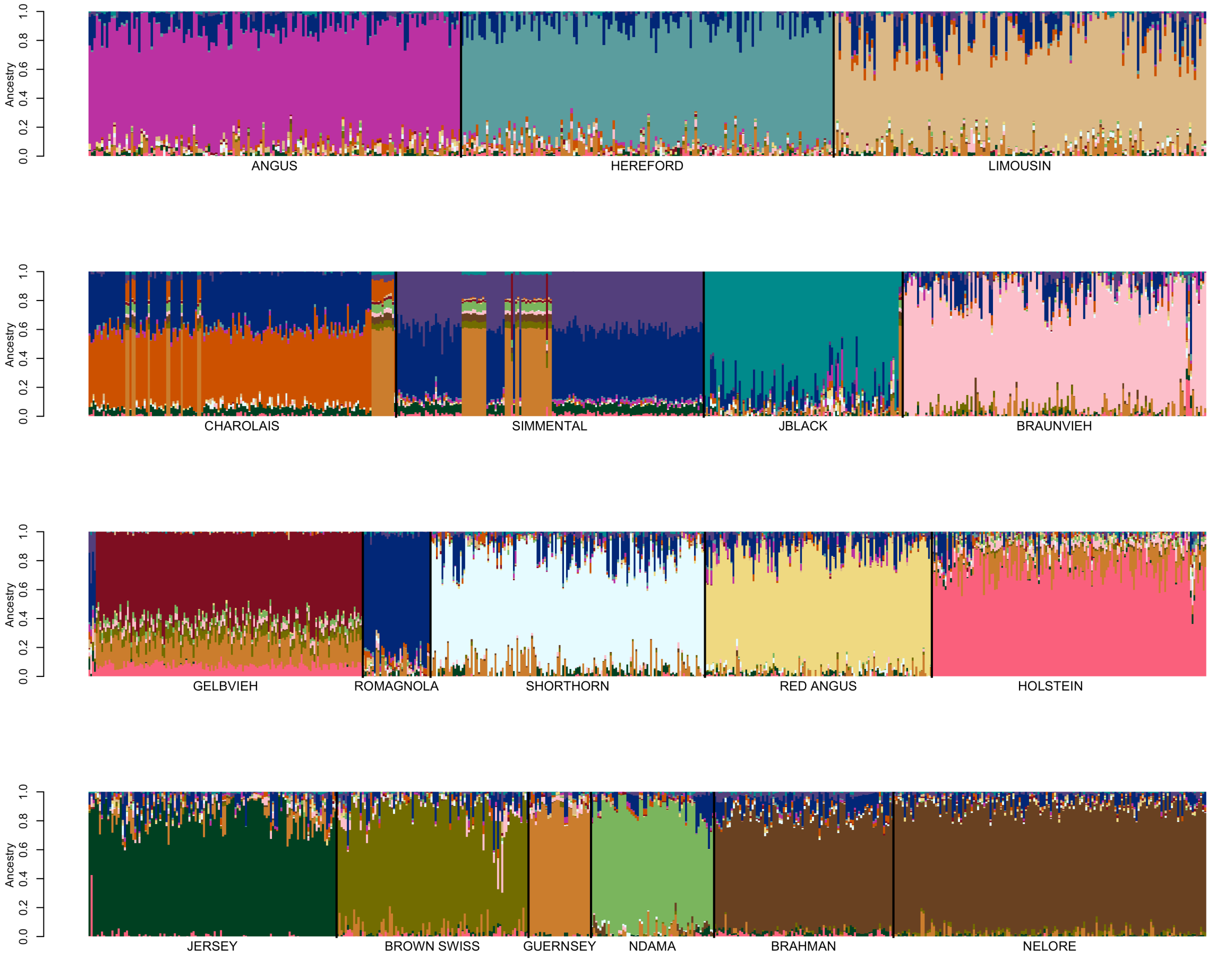

S10 Fig. SNPweights self-assignment analysis for the reference sample set containing ≤150 individuals per breed analyzed using the BC7K marker set.
