# Supplementary material for: CRUMBLER: A tool for the prediction of ancestry in cattle
Source: PLoS One. 2019 Aug 26;14(8):e0221471. doi: 10.1371/journal.pone.0221471 (PMC6709893; doi:10.1371/journal.pone.0221471)
Supplement: S12 Fig — (PDF) [file pone.0221471.s014.pdf]

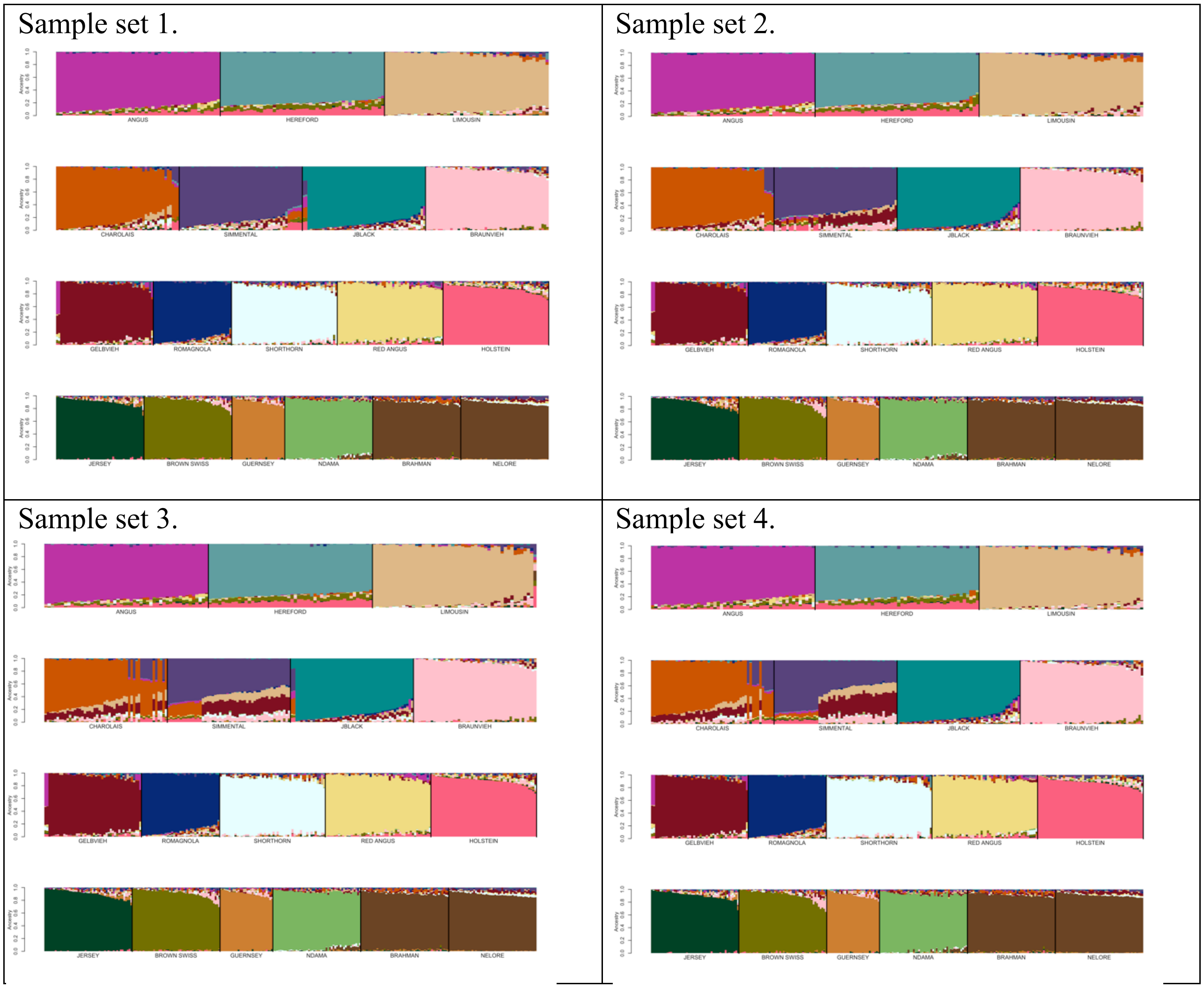

S12 Fig. SNPweights self-assignment analysis for the reference sample set containing ≤50 individuals per breed analyzed using the BC13K marker set. Reference breed panels were constructed by randomly sampling ≤50 individuals per breed until all individuals were represented in at least one set, resulting in 5 candidate reference sample sets (sample set 5 is shown in Fig. 4b).
