# Supplementary material for: CRUMBLER: A tool for the prediction of ancestry in cattle
Source: PLoS One. 2019 Aug 26;14(8):e0221471. doi: 10.1371/journal.pone.0221471 (PMC6709893; doi:10.1371/journal.pone.0221471)
Supplement: S13 Fig — (PDF) [file pone.0221471.s015.pdf]

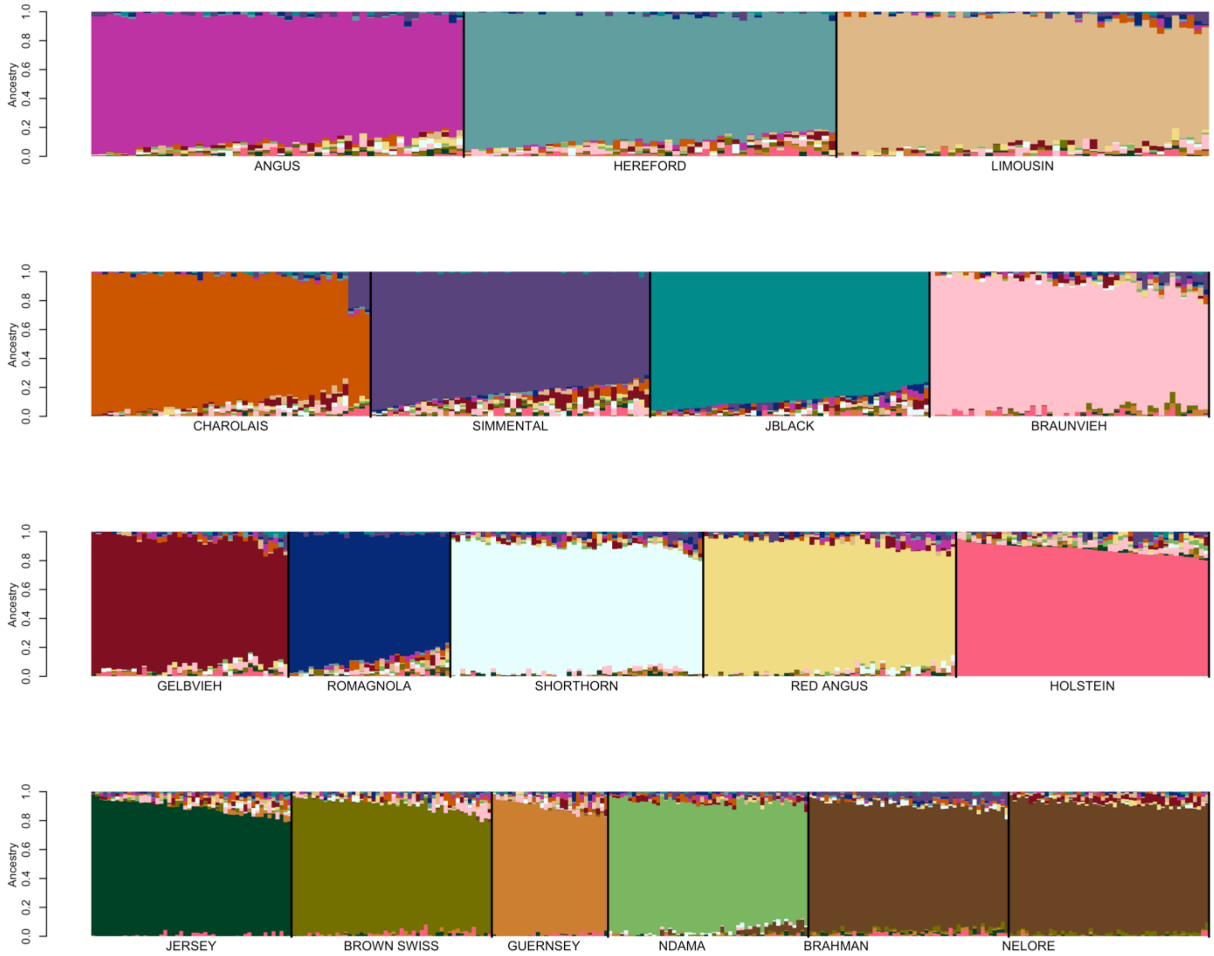

S13 Fig. SNPweights self-assignment analysis for the reference sample set with ≥80% ancestry to breed of registry and ≤50 individuals per breed using the BC7K marker set.
