# Supplementary material for: CRUMBLER: A tool for the prediction of ancestry in cattle
Source: PLoS One. 2019 Aug 26;14(8):e0221471. doi: 10.1371/journal.pone.0221471 (PMC6709893; doi:10.1371/journal.pone.0221471)
Supplement: S16 Fig — (PDF) [file pone.0221471.s018.pdf]

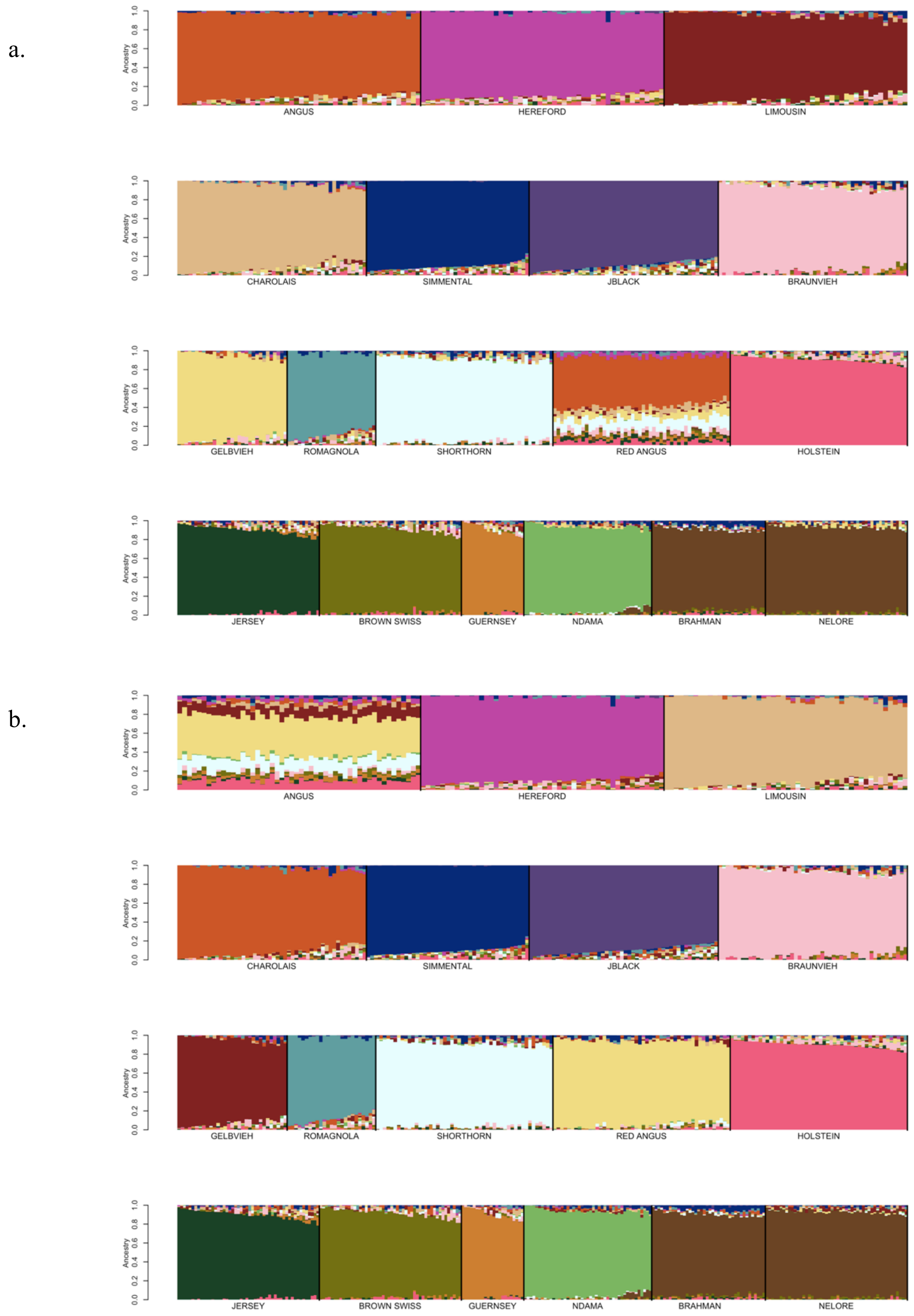

S16 Fig. SNPweights self-assignment analyses using a reference panel with ≤50 individuals per breed and sampling from the individuals with ≥85% assignment to their breed of registry but with (a) Red Angus or (b) Angus excluded from the reference panel. The Red Angus and Angus individuals in the reference panel were retained for ancestry estimation.
